# Low Incidence of HIV-1C Acquired Drug Resistance 10 Years after Roll-Out of Antiretroviral Therapy in Ethiopia: A Prospective Cohort Study

**DOI:** 10.1371/journal.pone.0141318

**Published:** 2015-10-29

**Authors:** Andargachew Mulu, Melanie Maier, Uwe Gerd Liebert

**Affiliations:** 1 Institute of Virology, Faculty of Medicine, Leipzig University, Leipzig, Germany; 2 Department of Microbiology, College of Medicine and Health Sciences, University of Gondar, Gondar, Ethiopia; University of Rome Tor Vergata, ITALY

## Abstract

The emergence of HIV-1 drug resistance mutations has mainly been linked to the duration and composition of antiretroviral treatment (ART), as well as the level of adherence. This study reports the incidence and pattern of acquired antiretroviral drug resistance mutations and long-term outcomes of ART in a prospective cohort from Northwest Ethiopia. Two hundred and twenty HIV-1C infected treatment naïve patients were enrolled and 127 were followed-up for up to 38 months on ART. ART initiation and patients’ monitoring was based on the WHO clinical and immunological parameters. HIV viral RNA measurement and drug resistance genotyping were done at baseline (N = 160) and after a median time of 30 (IQR, 27–38) months on ART (N = 127). Viral suppression rate (HIV RNA levels ≤ 400 copies/ml) after a median time of 30 months on ART was found to be 88.2% (112/127), which is in the range for HIV drug resistance prevention suggested by WHO. Of those 15 patients with viral load >400 copies/ml, six harboured one or more drug resistant associated mutations in the reverse transcriptase (RT) region. Observed NRTIs resistance associated mutations were the lamivudine-induced mutation M184V (n = 4) and tenofovir associated mutation K65R (n = 1). The NNRTIs resistance associated mutations were K103N (n = 2), V106M, Y181S, Y188L, V90I, K101E and G190A (n = 1 each). Thymidine analogue mutations and major drug resistance mutations in the protease (PR) region were not detected. Most of the patients (13/15) with virologic failure and accumulated drug resistance mutations had not met the WHO clinical and/or immunological failure criteria and continued the failing regimen. The incidence and pattern of acquired antiretroviral drug resistance mutations is lower and less complex than previous reports from sub Saharan Africa countries. Nevertheless, the data suggest the need for virological monitoring and resistance testing for early detection of failure. Moreover, adherence reinforcement will contribute to improving overall treatment outcomes.

## Background

The rapid scale-up of antiretroviral therapy (ART) in resource-limited countries has dramatically reduced HIV-related mortality and therefore improved quality of life [1, 2]. Contrary to the initial fears, a number of reports have documented successful short-term outcomes of ART [3, 4] due to many factors such as improved adherence [5–7]. Nevertheless, scaling up of ART programs have also led to the emergence of antiretroviral drug resistance [6]. The exclusive use of clinical and immunological parameters to initiate and monitor ART in this region and the use of drugs with low genetic barrier that only require single point mutation to confer resistance, such as abacavir (ABC), lamivudine (3TC), tenofovir (TDF) and didanosine (ddI) have been associated with the emergence and accumulation of HIV-1 drug-resistant variants [7–9]. Consequently, early virological failure remains undetected and patients continue to be on inadequate (or insufficient) regimen until either clinical or immunological failure occurs [8, 9]. In the meantime, resistance mutations may accumulate in the viral pool and in-turn limit future treatment options [10], enhance disease progression [11, 12] and may be transmitted to other individuals [10]. High level and complex profile of HIV-1 drug resistance mutations including Q151M and thymidine analogue mutations (TAMs) in individuals with first-line ART failure [8, 9, 13, 14, 15, 16] and increased prevalence of transmitted drug resistance (TDR) mutations have been reported in recent years [17, 18, 19, 20, 21], despite more promising initial observations from Africa [4, 6, 13].

In Ethiopia increasing prevalence of TDR with time after the roll-out of ART [18, 19], the late presentation of patients in the course of HIV infection and the use of WHO clinical and immunological parameter without HIV viral-load monitoring and drug resistance testing could be associated with delayed switching and consequent accumulation of resistance mutations, as it has been observed in other African countries [1, 8, 9]. This study therefore aimed to determine and characterize the incidence and pattern of acquired drug resistance mutations after a median time of 30 months (IQR, 27–38) on ART in a prospective cohort of HIV-1 subtype C infected patients with known baseline data [18].

## Methods

### Study design

Patient’s inclusion and exclusion criteria have already been described [18]. Briefly, HIV-1 chronically infected treatment naïve patients above 18 years of age and seeking care and treatment were consecutively recruited at Gondar University Hospital, Northwest Ethiopia in 2008/2009. Patients were excluded if they were pregnant or had taken single dose nevirapine (NVP) for prevention of mother to child transmission (PMTCT) or patients with known chronic illness or any previous ART use. In this prospective cohort study the long term clinical, immunological and virological outcomes of ART was examined and incidence of acquired drug resistance mutations was determined by the median time of 30 months on ART.

### Treatment and patients monitoring

Patients were evaluated with a standardized form at enrolment and diagnosed based on the WHO criteria of AIDS-defining conditions as described earlier [18]. The patients received care and treatment according to the national HIV treatment guideline and were monitored according to the WHO recommendations. First line ART included zidovudine (ZDV), 3TC, stavudine (D4T), nevirapine (NVP), and efavirenz (EFV), permitting four alternative treatment regimens: D4T+3TC+NVP; D4T+3TC+EFV; ZDV+3TC+NVP; or ZDV+3TC+EFV. For second line therapy, the nucleoside backbone waschanged to ABC, TDF, ddI or ZDV (if not used in first-line therapy); and, in addition, the non-nucleoside reverse transcriptase inhibitors (NNRTIs) NVP or EFV was replaced by one of the boosted protease inhibitors (PIs) lopinavir (LPV/r), saquinavir (SQV/r) or indinavir (IND/r). Although D4T has been omitted from ART recommendations because of its side effects in 2008, it is still used extensively in Ethiopia.

### Assessment of adherence

As per the routine clinical practice and the protocol of this cohort each patient underwent an adherence counselling before initiation of ART and at their health care visits every three months. Consecutively, self-reported adherence to ART was assessed by asking the last time the patient forgot to take therapy in four weeks time (response options include “never,” “yesterday,” “last week,” and “more than 1 week but less than three weeks ago”). Then, an estimate of patient's current adherence to antiretroviral therapy (as “optimal,” “suboptimal,” and “absent”) was made. Patients who reported that they had not forgotten a dose the day prior to their visit and never forget doses were categorized as optimal (theoretical adherence level of at least 95%) [5].

### Blood collection

Blood samples was collected in vacutainer tubes containing ethylene diamine tetraacetic acid (EDTA) at baseline, every 6 months (for CD4^+^ T cells count only) and at last by a median time of 30 months on ART (IQR, 27–38). Plasma was separated by centrifugation and stored at-40°C utile used.

### Laboratory investigations

Sample collection and preparation, CD4^+^ T cells count, HIV-1 RNA extraction, pol gene amplification and sequencing were done as described previously [18]. In brief, CD4^+^ T cells count was measured every 6 months using the FACSCount flow cytometer (Becton Dickinson, San Jose, CA, USA) following the manufacturer’s protocols. Immunological failure was defined as failure to achieve a CD4^+^ T cells gain of at least 50 cells above pre-therapy level or having an absolute CD4^+^ T cells count of less than 100 cells/mm^3^ after one year of therapy. HIV viral load was measured at baseline (N = 220) and after a median time of 30 months on ART (N = 127) using Abbott m2000rt Quantitative RealTime HIV-1 assay (Abbott Molecular, Des Plaines, IL, USA) with a lower detection limit of 40 copies/ml.

Although, WHO defines virological failure using viral load cut-offs of >1000 copies/ml based on two consecutive viral load measurement after 3 months with adherence support, there is no standardized reporting of virologic failure since 3 months may not enough for full viral suppression, and since resistance mutations could be detected in samples with VL of as low as 300 copies/mL and not in patients with viral load of >1000 copies/ml. Thus, in this study, virological failure is defined as HIV RNA > 400 copies/mL in single plasma sample and viral load between 40 and 400 copies/ml was defined as low-level viraemia (LLV). HIV genotyping for detection of drug resistance was done at baseline and after a median time of 30 months on ART (N = 127). Viral cDNA was amplified by nested PCR using outer primers yielding a 1757 base pairs (bp) amplicon and subsequently by the inner primers yielding a 1389 bp amplicon covering the entire protease (PR) and partial (76%) reverse transcriptase (RT) enzymes following an in-house protocol. The amplified HIV-1 viral gene was sequenced using BigDye Terminator Cycle Sequencing kit (Applied Biosytems, Waltham, MA, USA). Genotypic drug resistance mutations were interpreted according to the Stanford University drug resistance database (http://hivdb.stanford.edu) and the 2014 IAS mutation list [22]. HIV-1 subtype was determined by using the REGA HIV-1 Automated Subtyping Tool version 2 (http://www.bioafrica.net). Nucleotide sequences are deposited in National Centre for Biotechnology Information (NCBI), USA GenBank (Accession Number before initiation of ART: KF026059-KF026220; By the end of 30 months on ART: KT020928-KT020935).

### Statistical analysis

To identify potential risk factors associated with virologic failure, a bivariable and multivariable logistic regression analysis were conducted by the end of 30 months. Variables included were age, sex, baseline CD4^+^ T cells count, baseline HIV viral load, WHO clinical stage, baseline drug resistance mutations, level of adherence and the type of ART (NVP versus EFV containing). Differences between viraemic and non-viraemeic groups were tested using the Pearson Chi-square test, the student’s t-test and the Fisher’s exact test, as appropriate. A P-value of less than 0.05 was considered statistically significant.

### Ethical approval

The study protocol and design including the consent procedures were approved by the University of Gondar Ethical Review Committee (RPO/55/291/00). Patients were managed following the national guideline. Written informed consent was obtained from all study subjects and documented in Research Office of the University.

## Results

### Cohort characteristics

**Fig 1:**
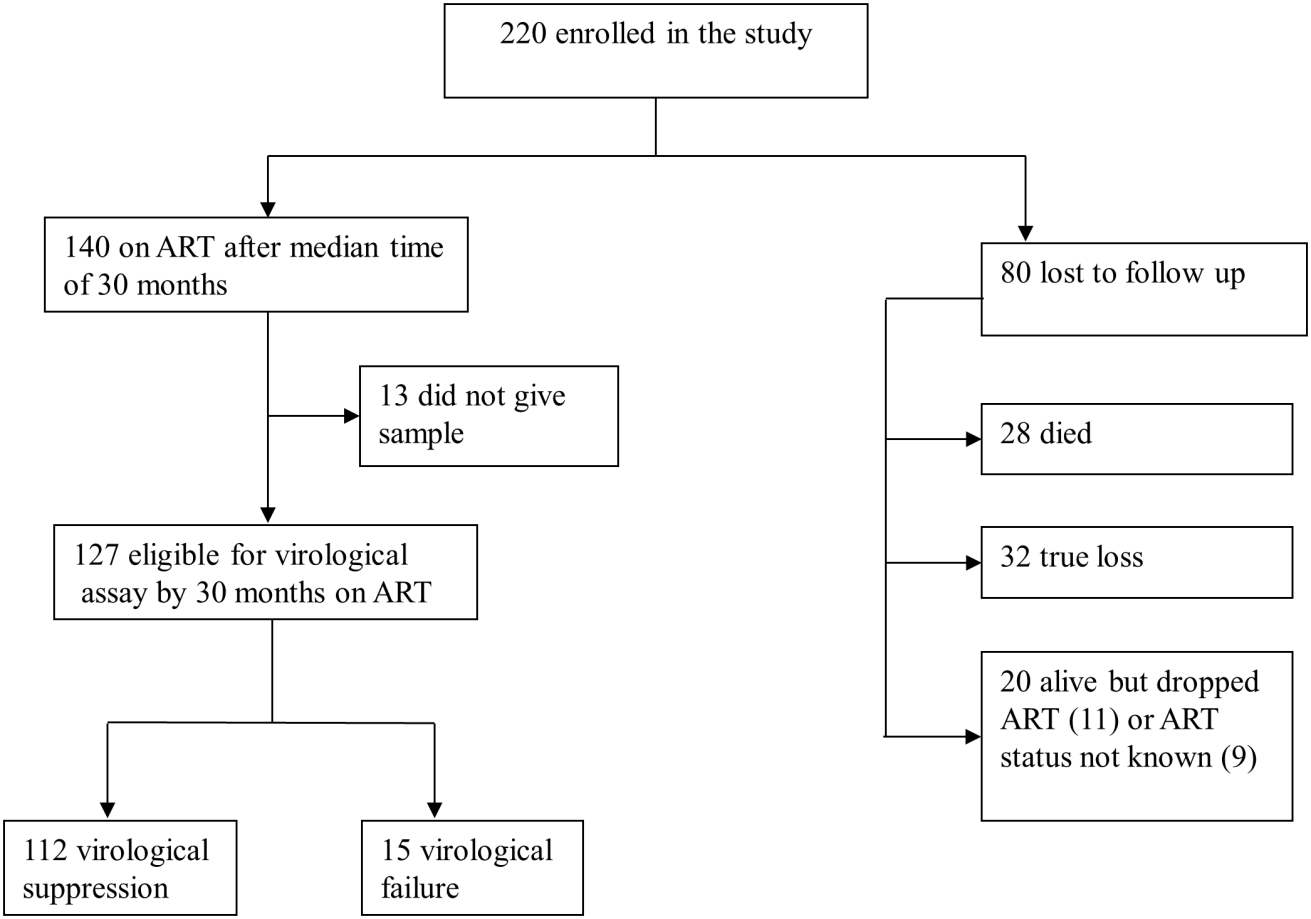
Cohort profile. Of the 220 subjects enrolled in the study 63.6% (140/220) were on ART and blood collection for HIV DR was successful in 127 patients; Confirmed death: 12.7% (28/220); overall death including true loss: 27.3% (60/220); True loss: 14.5% (32/220).

The baseline characteristics of the patients at enrolment were described earlier [18]. In brief, HIV genotypic drug resistance testing was performed in 160 pre-ART plasma samples and on the basis of the WHO transmitted drug resistance surveillance mutations list (S1 Table), transmitted HIV drug resistance mutations were identified in 21 (13.12%) of the samples. All except to 2 isolates were found to be HIV-1C. Of 220 patients enrolled, 140 patients were on ART by median time of 30 months (IQR, 27–38) but this study has end viral load result for 127 patients. Thus, the results below are for these 127 patients. The cohort profile is summarized in Fig 1.

### Virological outcomes

By on treatment analysis (including patients with both baseline and median time of 30 months virological data), 88% (112/127) of the patients achieved viral suppression with HIV RNA levels ≤ 400 copies/ml of whom 74% (83/112) had undetectable HIV RNA level (Table 1). The median (IQR) baseline HIV viral load for 127 patients was 27, 137 copies/ml (4273–133319). Among the 15 patients with viral loads of above 400 copies/ml, 11 met the WHO virological failure criteria of viral loads of >1000 copies/ml. However, most of the patients (13/15) with virological failure and accumulated drug resistance mutations had not met the WHO clinical and immunologic failure criteria and continued the failing regimen. Moreover, in 2 patients with viral loads < 5000 copies/ml, drug resistance associated mutations were detected. In two patients with HIV RNA of 21, 878 and 97, 724 copies/ml, accumulated drug resistance mutations were not observed (Table 1). There was no significant difference in the proportion of patients with baseline CD4^+^ T cells counts of > 200 cells/mm^3^ with virological failure compared with those with a baseline CD4^+^ T cells count of ≤ 200 cells/mm^3^. The median HIV RNA level among viremic patients at 30 months was not significantly different among those on NVP and EFV (906 copies/ml (525–42986) versus 2198 copies/ml (1545–23389); P > 0.05).

**Table 1:** Characteristics of the patients classified as non viremic or viremic after a median time of 30 months on ART (upper) and median CD4 T cell and RNA values (lower).

Characteristics	Non viremic* (n = 112)	Viremic^¶^ (n = 15)
Gender		
Male	48 (42.8)	6 (40)
Female	64 (57.2)	9 (60)
WHO stage at ART initiation		
Stage I/II	40 (35.7)	4 (26.7)
Stage III/IV	72 (64.3)	11 (73.3)
CD4^+^ T cell count at baseline		
<200 cells/ mm^3^	62 (55.4)	7 (46.7)
≥200 cells/ mm^3^	50 (44.6)	8 (53.3)
CD4^+^ T cell counts at 30 months of ART		
<200 cells/ mm^3^	6 (5.4)	3 (20.0)
≥200 cells/ mm^3^	106 (94.6)	12 (80.0)
First line ART		
3TC + D4T + NVP	52 (46.4)	6 (40.0)
3TC + D4T + EFV	21 (18.8)	2 (13.3)
3TC + AZT + NVP	27 (24.1)	3 (20.0)
3TC + AZT + EFV	12 (10.7)	4 (26.7)
Median (IQR) age [Years]	33 (18–62)	33.8 (23–58)
Median (IQR) CD4^+^ T cells/mm^3^at baseline	204 (26–203)	170 (75–229)
Median (IQR)CD4^+^T cells/mm^3^ at 30m of ART	365 (259–434)	387 (299–426)
Median (IQR) HIV RNA level at baseline	57328 (20481–172442)	19059 (5754–51833)
HIV RNA at 30 months of ART		
Undetectable	83 (74.1)	0
1–40	6 (5.4)	0
41–400	23 (20.5)	0
401–5000	0	5 (33.3)
>5000	0	10 (66.7)
Median follow up time (IQR) [months]	30.0 (26–36)	31.0 (25–35)

* HIV RNA < 400 copies/ml;

^¶^HIV RNA ≥ 400 copies/ml;

3TC (lamiduvine), D4T (stavudine), ZDV (zidovudine), TDF (tenofovir), EFV (efavirenz), NVP (nevirapine); IQR (Inter quartile range)

### HIV genotyping drug resistance

Genotyping was successfully determined in 8 of the 15 patients with HIV RNA ≥ 400 copies/ml. Amplification failures were due to low viral loads (ranging from 442 to 1886 copies/ml) probably representing virological blips. Accumulation of drug resistance mutations in RT region selected during treatment was observed in 6 of 8 patients (Table 2). Briefly, 6/8 patients on 3TC developed resistance to 3TC, 2/3 patients on EFV developed resistance to EFV and NVP, 3/5 patients on NVP developed resistance to NVP and 2/5 patients on NVP developed resistance to EFV within 30 months of ART exposure. The most frequent NRTIs resistance associated mutations were the lamivudine-induced M184V mutation (n = 4) and K65R (n = 1). The NNRTIs resistance associated mutations V106M and K103N conferring resistance to EFV and NVP were found in 1 and 2 patient/s, respectively. Other NNRTIs associated mutations Y181S, Y188L, V90I, K101E and G190A were observed in one patient each. Dual-class resistance to NRTIs and NNRTIs were detected in 4 patients with frequent combination of M184V and NNRTI resistance associated mutations. Despite the extensive use of thymidine analogues (TA) like AZT or D4T, TAMs and mutation at codon 151 (Q151M) were not observed. Moreover, major drug resistance mutations on PR region were not detected. However, in all the 8 patients, naturally occurring minor mutations/polymorphic changes at PR region (positions M36I, R41K, H69K, L89M, and I93L) were observed. These mutations already existed in the baseline sequence data.

**Table 2:** Acquired antiretroviral drug resistance mutations among subtype C Ethiopian patients after a median time of 30 months on ART.

		CD4^+^ T cells		HIV RNA		Baseline ART	Time on ART	NRTI		NNRTI	
ID	Age/Gender	before	after	before	after			mutation	resistant	mutation	resistant
5622–2	51/M	250	391	26,915	19,952	3TC+D4T+EFV	25	M184V	3TC, FTC	K103N	EFV, NVP
5593–2	28/M	401	449	14,791	2,511	3TC+D4T+EFV	35	M184V	3TC, FTC	K103N	EFV, NVP
										V106M	EFV, NVP, ETR
5685–2	25/F	229	393	26, 9153	91,201	3TC+D4T+NVP	29	None	-	Y181SY	NVP
5524–2	38/M	8	276	9,120	91,201	3TC+D4T+NVP	33	K65R	3TC,DDI,FTC, TDF	Y188L	EFV, NVP
5603–2	25/F	32	270	158	4,786	3TC+D4T+NVP	29	M184V	3TC, FTC	V90I	EFV, NVP, ETR
										K101E	EFV, NVP, ETR
										G190A	EFV, NVP, ETR
5476–2	29/F	199	281	26,302	53,703	3TC+D4T+EFV	35	M184V	3TC, FTC	None	-
5708–2	38/F	274	319	1,071	26,915	3TC+D4T +NVP	29	None	-	None	-
5591–2	30/M	32	299	5,754	97,923	3TC+D4T+NVP	31	None	-	None	-
5542–2	42/F	75	474	33,414	442	3TC+D4T+NVP	28	None	-	None	-
5776–2	40/M	191	426	80,013	553	3TC+D4T+NVP	32	None	-	None	-
5666–2	38/F	157	387	51,833	889	3TC+D4T+NVP	36	None	-	None	-
5684–2	50/F	95	416	103,443	1,570	3TC+D4T+EFV	34	None	-	None	-
5726–2	40/F	170	383	19,059	1,886	3TC+D4T+EFV	32	None	-	None	-
5573–2	51/M	112	463	11,433	871	3TC+D4T+NVP	30	None	-	None	-
5635–2	35/M	211	301	5,635	1,471	3TC+D4T+EFV	29	None	-	None	-

Age: in years; F: Female, M: Male; CD4^+^ T in cells/mm^3^; HIV RNA in copies/ml; Before: Before ART (baseline); After: After initiation of ART; Time on ART in months; NRTI (Nucleoside RT inhibitors): 3TC (lamiduvine), ddI (didanosine), d4T (stavudine), FTC (emtricitabine), TDF (tenofovir), ZDV (zidovudine); NNRTI (non-nucleoside RT inhibitors): EFV (efavirenz), ETR (etravirine), NVP (nevirapine); Amino acids: A (alanine), E (glutmatate), G (glucine), K (lysine), L (leucine), M (methionine), N (asparganine), S (serine), V (valine), Y (tyrosine)

### Clinical and immunological outcomes

**Fig 2:**
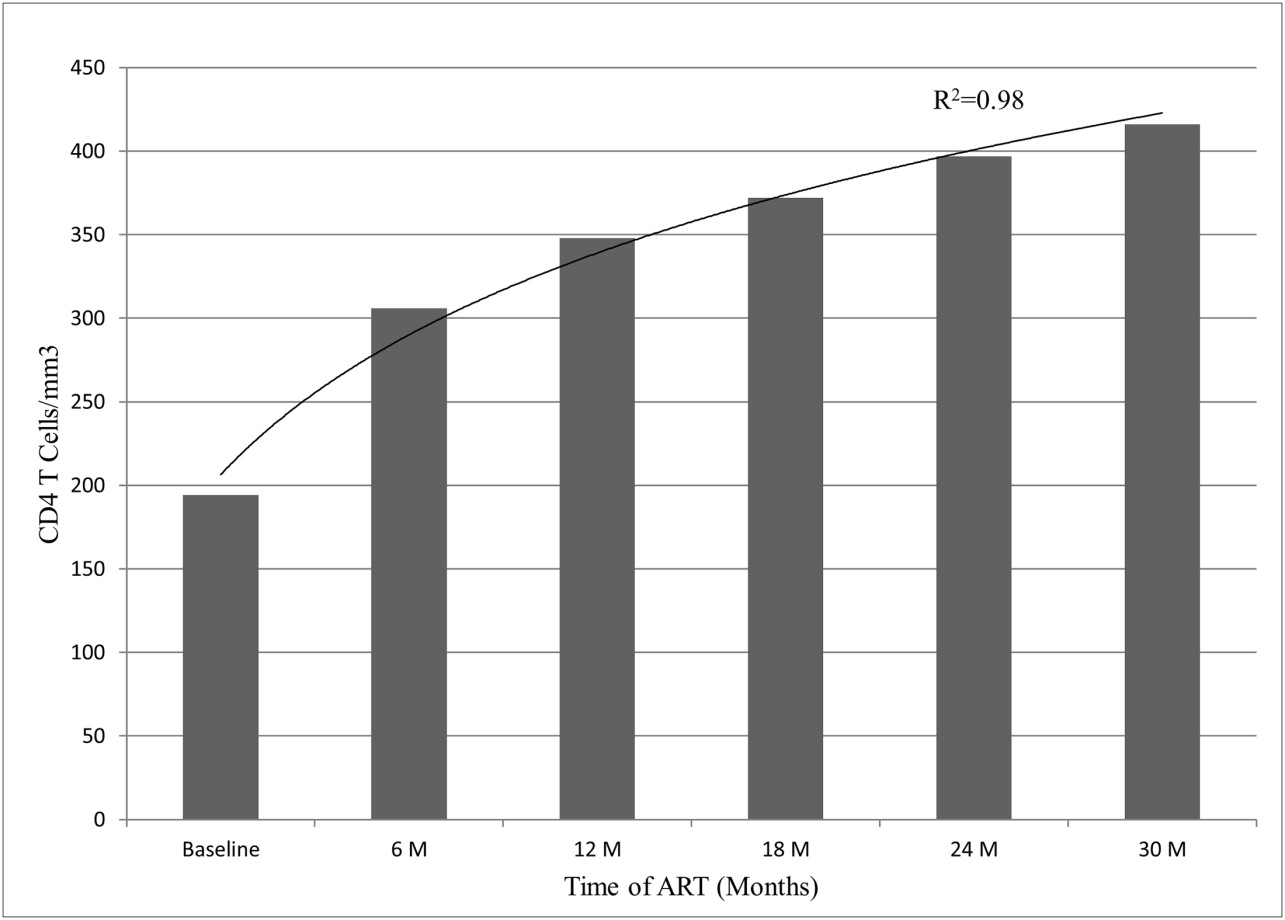
Immunological restoration among HIV-1C Ethiopian patients (N = 127 at each time point) during 30 months of ART.

By the median time of 30 months on ART, 18 out of 127 patients (14.2%) were non-adherent at least once. The main reasons for non-adherence were health related problems (9/18) and drug side effects (5/18). Accordingly, treatment switching was made (3 due to clinical failure, 6 due to immunological failure and 4 due to drug associated toxicity: 3 patients stopped AZT due to anaemia, 1 stopped EFV due to neuropsychiatric disorder and 1 stopped d4T due to neuropathy and lipodystrophy) at a median time of 61 days after initiation of ART. All the patients were on appropriate ART regimen as per the National ART guideline and were clinically asymptomatic. The median CD4^+^ T cells count of the 127 patients at baseline was 194 (IQR, 25–229) cells/mm^3^ and progressively increased over time (Fig 2). The rate of CD4^+^ T cells count increased significantly in the first 12 months period (+183 cells/mm^3^) exceeding the rate of the following 24 months (+81 cells/mm^3^, P <0.01, 95% CI, 69–91). The rate of CD4^+^ T cells recovery was not different among patients who were taking NVP versus EFV based regimen (+211 vs + 217 cells/mm^3^; P = 0.14).

### Factors associated with virological failure

In both bivariable and multivariable analyses, baseline severe immunosuppression (defined as CD4^+^ T cells count of < 50 cells/mm^3^) and baseline HIV RNA of >100,000 copies/ml were not identified as risk factors for virological failure at 30 months. The presence of baseline transmitted drug resistance mutations was not a risk factor for virological failure by the median time of 30 months on ART, either. However, 14 of 21 patients with baseline transmitted drug resistance associated mutations were lost to follow-up and only 7 out of 21 achieved viral suppression. Other demographic predicting factors (age, sex, WHO clinical stages, type of ART regimen) were also not significant risk factors for virological failure at 30 months (Table 3). However, treatment non-adherence was identified as significant risk factor for virological failure at 30 months (OR 5.11:95%CI; 2.11–21.00, P = 0.02).

**Table 3:** Factors associated with virological failure by 30 months of ART.

Variables	Crude		Adjusted	
	OR (95% CI)	P value	OR (95% CI)	P value
Gender				
Male	Reference			
Female	1.23 (0.56–2.57)	0.09	1.37 (0.65–3.17)	0.12
Age (years)	0.91 (0.81–1.12)	0.18	0.94(0.83–1.17)	0.27
Baseline WHO clinical stage				
Stage I	Reference			
Stage II	2.13(0.63–6.73)	0.13	1.89(0.54–6.17)	0.19
Stage III	1.38 (0.48–4.72)	0.17	3.81 (0.81–4.12)	0.07
Stage IV	0.88 (0.31–4.63)	0.22	1.28 (1.74–9.19)	0.8
Baseline CD4^+^ T cell counts*				
>200	Reference			
50–200	1.49 (0.19–4.13)	0.66	1.58(0.43–5.34)	0.34
<50	1.74 (0.37–4.39)	0.69	1.83(0.44–5.76)	0.39
Baseline HIV RNA level**				
<10, 000	Reference			
10, 001–100, 000	1.31(0.59–4.79)	0.33	2.18 (0.79–5.94)	0.36
>100, 000	0.46(0.11–6.70)	0.13	0.93 (0.62–7.21)	0.12
First line ART				
NVP containing regimen	Reference			
EFV containing regimen	2.33 (0.55–3.37)	0.4	1.36 (0.93–3.17)	0.31
Transmitted drug resistance				
No	Reference			
Yes	1.17 (0.29–3.88)	0.35	1.77 (0.43–5.41)	0.08
Level of adherence				
Optimal	Reference			
Sub-optimal	1.48 (0.66–9.49)	0.15	2.83 (0.73–9.03)	0.6
Absent	4.78 (2.53–11.17)	0.01	5.11 (2.11–21.00)	0.02

*CD4^+^ T cells count in cells/mm^3^;

**HIV RNA level: copies/ml

## Discussion

This is the first report describing the incidence and pattern of acquired antiretroviral drug resistance mutations among HIV-1C infected patients in a prospective cohort study from the Horn of Africa with in a median time of 30 months on ART. The 12% virological failure (≥400 copies/ml) in the current study is comparable with recent cross-sectional studies from the region [4, 8, 13, 23] and WHO suggested targets of virological suppression were achieved in at least 70% of patients 12 months after ART initiation [17]. However, recent studies from some African countries had reported a high level of virological failure after a weighted median time of 25 months on ART [13, 24, 25]. Thus, compared with the few available data on the long term effect of ART our finding showed low rate of virological failure which could be due to the optimal adherence level of the patients (85.8%) and in the setting [5] and suggests that ART in the setting can sustain virological efficacy for a substantial length of time if an optimal adherence on treatment is achieved. However, these results may not fully represent other public HIV clinics in Ethiopia.

The emergence and accumulation of acquired antiretroviral drug resistance mutations in 6 of 8 patients (Table 2) reflect the expected pattern of acquired drug resistance while using the standard first line ART attributed to the 3TC, NVP or EFV pressure. The findings were similar to that of subtype C sub Saharan African isolates [3, 13] and other subtypes in resource-limited settings [26, 27]. The K65R known to be selected faster in subtype C was observed in one patient who has been taking 3TC, D4T and EFV. This is very low compared to similar studies among subtype C isolates from six sub Saharan African countries [24] but consistent with recent study from South Africa [28]. This could be due to the recent availability of TDF in Ethiopia [4, 18] and because of the fact that none of the patients were under TDF. Among the NNRTIs mutations, K103N, V106M, and G190A conferring resistance to EFV and NVP; EFV, NVP and ETR; and EFV and NVP, respectively were observed in 2 and 1 patient each similar to recent data from various African countries [4, 13, 18, 25]. Four out of six patients failed as a result of M184V and NNRTIs mutation. Five out of 6 patients had NNRTIs mutations probably suggesting that resistance to NNRTIs are the initial event in failure of regimens containing EFV or NVP in the setting. Despite the extensive use of thymidine analogues like AZT or D4T in Ethiopia and unlike previous similar studies [3, 13, 27–29], TAMs characterized by the mutations M41L, L210W, T215Y (TAM-path 1) and D67N, K70R, T215F, K219Q/E (TAM-path 2) were lacking in the current study indicating a recent period of virological failure. This also supports previous reports that mutations correlated with TAMs pathways are infrequent in non B subtypes compared to subtype B [4, 17–25, 26–29]. The absence of major drug resistance mutations in PR region might be a reflection of late introduction and limited access of this drug class in the country [4, 18].

Despite the small number of sequences, the spectrum of drug resistance mutations observed in this study was not complex which is consistent with a recent study from Rwanda [30]. However, the occurrence of virological failure and resistance mutations in most of the patients without reaching the WHO clinical and immunological criteria that presently apply to Ethiopia and other developing countries suggests the need for virological monitoring and targeted drug resistance testing in the region. These patients also continued the failing regimen which would limit future treatment option and may result in deleterious consequences if transmission to newly infected individuals occurs. The low level viraemia (40–400 copies/ml) observed in a single sample of 23 clinically asymptomatic patient with immune recovery, supports previous findings that reported transient episodes of low level viraemia do not generally reflect negative clinical consequence [4, 31, 32] and show the presence of virological blips rather than virological failure. However, some may remain suppressed and others may develop virological failure if tested later.

In this prospective cohort, progressive immune restoration was observed over time similar to previous reports [4, 33–34]. It is, to be noted however, that after nearly 3 years of ART about one fifth of the patients remained below the lower threshold level (less than 350 CD4^+^ T cells/mm^3^). This could be due to the natural low CD4^+^ T cells count among Ethiopians [4, 35, 36], late ART initiation with low baseline CD4^+^ T cells count, slow recovery rate of CD4^+^ T cells among Africans [37] or may also be due to immune activation which results in CD4^+^ T cells count depletion independent of HIV RNA load [38, 39].

In conclusion, despite the obvious small number of sequences the findings suggest that in resource limited settings ART can sustain virological efficacy for a substantial length of time and also enhance immunological recovery if supplemented with adherence counselling ensuring an optimal adherence level. However, the 12% virological failure and resistance mutations observed in the absence of the WHO clinical and immunological criteria that presently apply to developing countries suggest the need for affordable virological monitoring and targeted drug resistance testing assays in the region.

## Supporting Information

S1 Table Baseline transmitted drug resistance in the RT gene among chronically infected patients from Northwest Ethiopia. Reprinted from BMC Infect Dis. 2014 Mar 22; 14:158. doi: 10.1186/1471-2334-14-158.
(PDF)

## References

[1] Gilks CF, Crowley S, Ekpini R, Gove S, Perriens J, Souteyrand Y, et al The WHO public-health approach to antiretroviral treatment against HIV in resource-limited settings. Lancet 2006 8 5; 368(9534). 10.1016/S0140-6736(06)69158-7 16890837

[2] World Health Organization. Towards universal access: scaling up priority HIV/AIDS interventions in the health sector (2010): progress report. Available: http://www.who.int/hiv/pub/2010progressreport/report/en/index.html

[3] Ivers LC, Kendrick D, Doucette K. Efficacy of antiretroviral therapy programs in resource-poor settings: a meta-analysis of the published literature. Clin Infect Dis 2005 7 15; 41(2). 10.1086/431199 15983918

[4] Mulu A, Liebert UG, Maier M. Virological efficacy and immunological recovery among Ethiopian HIV-1 infected adults and children. BMC Infect Dis 2014 1 14; 14(28). 10.1186/1471-2334-14-28 PMC3900473 24422906

[5] Tessema B, Biadglegne F, Mulu A, Getachew A, Emmrich, Sack U,. Magnitude and determinants of non-adherence and non-readiness to highly active antiretroviral therapy among people living with HIV/AIDS in Northwest Ethiopia: a cross—sectional study. AIDS Res Ther 1 14; 7 (2). 10.1186/1742-6405-7-2 PMC2845546 20180959

[6] Hamers RL, Derdelinckx I, van Vugt M, Stevens W, Rinke de Wit TF, Schuurman R, et al The status of HIV-1 resistance to antiretroviral drugs in sub-Saharan Africa. Antivir Ther 2008; 13(5). 18771046

[7] Gupta RK, Hill A, Sawyer AW, Cozzi-Lepri A, von Wyl V, Yirly S, et al Virological monitoring and resistance to first-line highly active antiretroviral therapy in adults infected with HIV-1 treated under WHO guidelines: a systematic review and meta-analysis. Lancet Infect Dis 2009 7; 9(7). 10.1016/S1473-3099(09)70136-7 19555900

[8] Hosseinipour MC, van Oosterhout JJ, Weigel R, Phiri S, Kamwendo D, Parkin N, et al The public health approach to identify antiretroviral therapy failure: high-level nucleoside reverse transcriptase inhibitor resistance among Malawians failing first-line antiretroviral therapy. AIDS2009 6 1; 23(9). 10.1097/QAD.0b013e32832ac34e PMC2896488 19417582

[9] Sigaloff KC, Hamers RL, Wallis CL, Kityo C, Siwale M, IVe P, et al Unnecessary antiretroviral treatment switches and accumulation of HIV resistance mutations; two arguments for viral load monitoring in Africa. J Acquir Immune Defic Syndr 2011 9 1; 58(1). 10.1097/QAI.0b013e318227fc34 21694603

[10] Kantor R, Shafer RW, Follansbee S, Taylor J, Shilane D, Hurley L, et al Evolution of resistance to drugs in HIV-1-infected patients failing antiretroviral therapy. AIDS 2004 7 23; 18(11). 10.1097/01.aids.0000131358.29586.6b PMC2547474 15238768

[11] Hogg RS, Bangsberg DR, Lima VD, Alexander C, Bonner S, Yip B, et al Emergence of drug resistance is associated with an increased risk of death among patients first starting HAART. PLoS Med 2006 9; 3(9). 10.1371/journal.pmed.0030356 PMC1569883 16984218

[12] Kozal MJ, Hullsiek KH, Macarthur RD, Berg-Wolf Mv, Peng G, Xiang Y, et al The Incidence of HIV drug resistance and its impact on progression of HIV disease among antiretroviral-naive participants started on three different antiretroviral therapy strategies. HIV Clin Trials 2007 Nov-Dec; 8(6). 10.1310/hct0806-357 18042501

[13] Barth RE, van der Loeff MF, Schuurman R, Hoepelman AL, Wensing AM. Virological follow-up of adult patients in antiretroviral treatment programmes in sub-Saharan Africa: a systematic review. Lancet Infect Dis 2010 3; 10(3). 10.1016/S1473-3099(09)70328-7 20185094

[14] Marconi VC, Sunpath H, Lu Z, Gordon M, Koranteng-Apeagyei K, Hompton J, et al Prevalence of HIV-1 drug resistance after failure of a first highly active antiretroviral therapy regimen in KwaZulu Natal, South Africa. Clin Infect Dis 2008 5 15; 46(10). 10.1086/587109 PMC2692213 18419495

[15] Wallis CL, Mellors JW, Venter WD, Sanne I, Stevens W. Varied patterns of HIV-1 drug resistance on failing first-line antiretroviral therapy in South Africa. J Acquir Immune Defic Syndr 2010 4 1; 53(4). 10.1097/QAI.0b013e3181bc478b 19801944

[16] Koyalta D, Charpentier C, Beassamda J, Rey E, Si-Mohamed A, Djemadji-Oudjeil N, et al High Frequency of Antiretroviral Drug Resistance among HIV-Infected Adults Receiving First-Line Highly Active Antiretroviral Therapy in N’Djamena, Chad. Clin Infect Dis 2009 7 1; 49(1). 10.1086/599611 19480574

[17] World Health Organization (2012) WHO HIV Drug Resistance Report 2012 (Available: http://apps.who.int/iris/bitstream/10665/75183/1/9789241503938_eng.pdf)

[18] Mulu A, Lange T, Liebert UG, Maier M. Clade homogeneity and Pol gene polymorphisms in chronically HIV-1 infected antiretroviral treatment naive patients after the roll out of ART in Ethiopia. BMC Infect Dis 2014 3 22;14:158 doi: 10.1186/1471-2334-14-158 24655349 10.1186/1471-2334-14-158 PMC3976149

[19] Huruy K, Maier M, Mulu A, Liebert UG. Limited increase in primary HIV-1C drug resistance mutations in treatment naïve patients in Ethiopia. J Med Virol 2015 6;87(6). 10.1002/jmv.24110 25649964

[20] Kiwelu IE, Novitsky V, Kituma E, Margolin L, Baca J, Manongi R, et al HIV-1 pol diversity among female bar and hotel workers in Northern Tanzania. PLoS One 2014 7 8; 9(7). 10.1371/journal.pone.0102258 PMC4087014 25003939

[21] Hamers R, Sigaloffa K, Kityob C, Mugyenyib P and de Wit TF. Emerging HIV-1 drug resistance after roll-out of antiretroviral therapy in sub-Saharan Africa. Curr Opin HIV AIDS 2013 1; 8(1). 10.1097/COH.0b013e32835b7f94 23143140

[22] Wensing AM, Calvez V, Günthard HF, Johnson VA, Paredes R, Pillay D, et al Update of the Drug Resistance Mutations in HIV-1: International AIDS Society–USA. Top Antivir Med 2014 Jun-Jul;22(3). PMC4392881 25101529

[23] Abdissa A, Yilma D, Fonager J, Audelin AM, Christensen LH, Olsen MF, et al Drug resistance in HIV patients with virological failure or slow virological response to antiretroviral therapy in Ethiopia. BMC Infect Dis 2014 4 4; 14 (181). 10.1186/1471-2334-14-181 PMC4234735 24708645

[24] Muwonga J, Edidi S, Butel C, Vidal N, Monleau M, Okenge A, et al Resistance to Antiretroviral Drugs in Treated and Drug-Naive Patients in the Democratic Republic of Congo. J Acquir Immune Defic Syndr 2011 7 1;57 Suppl 1:S27–33. doi: 10.1097/QAI.0b013e31821f596c 21857282 10.1097/QAI.0b013e31821f596c

[25] Hamers RL, Sigaloff KC, Wensing AM, Wallis CL, Kityo C, Siwale M, et al Patterns of HIV-1 Drug Resistance after first-line antiretroviral therapy (ART) failure in 6 sub-Saharan African countries: Implications for second-line ART strategies. Clin Infect Dis 2012 6; 54(11). 10.1093/cid/cis254 22474222

[26] Bourgeois A, Laurent C, Mougnutou R, Nkoue N, lactuock B, Ciaffi L, et al Field assessment of generic antiretroviral drugs: a prospective cohort study in Cameroon. Antivir Ther 2005; 10(2). 15865228

[27] Ferradini L, Jeannin A, Pinoges L, Izopet J, Odhiambo D, Mankhambo L, et al Scaling up of highly active antiretroviral therapy in a rural district of Malawi: an effectiveness assessment. Lancet 2006 4 22; 367(9519). 10.1016/S0140-6736(06)68580-2 16631912

[28] Wallis CL, Papathanasopolous MA, Fox M, Conrade, Ive P, Zeinecker J, et al Low rates of nucleoside reverse transcriptase inhibitor resistance in a well-monitored cohort in South Africa on antiretroviral therapy. Antivir Ther 2012; 17(2). 10.3851/IMP1985 PMC3600633 22293461

[29] Kouanfack C, Montavon C, Laurent C, Aghokeng A, Kenfack A, Bourgeois A, et al (2009) Low levels of antiretroviral resistant HIV infection in a routine clinic in Cameroon that uses the World Health Organization (WHO) public health approach to monitor antiretroviral treatment and adequacy with the WHO recommendation for second-line treatment. Clin Infect Dis 2009 5 1; 48(9). 10.1086/597779 19320592

[30] Rusine J, Asiimwe-Kateera B, van de Wijgert J, Boer KR, Mukantwali E, Karita E, et al Low Primary and Secondary HIV Drug-Resistance after 12 Months of Antiretroviral Therapy in Human Immune-Deficiency Virus Type 1 (HIV-1)-Infected Individuals from Kigali, Rwanda. PLoS ONE 2013 8 12; 8(8). 10.1371/journal.pone.0064345 PMC3741294 23950859

[31] Doyle T and Geretti AM. Low-level viraemia on HAART: significance and management. Curr Opin Infect Dis 2012 2;25(1). 10.1097/QCO.0b013e32834ef5d9 22156900

[32] Geretti AM, Smith C, Haberl A, Garcia-Diaz A, Nebbia G, Johnson M, et al Determinants of virological failure after successful viral load suppression in first-line highly active antiretroviral therapy. Antivir Ther 2008; 13(7). 19043927

[33] Coetzee D, Hildebrand K, Boulle A, Maartens G, Louis F, Labatala V, et al Outcomes after two years of providing antiretroviral treatment in Khayelitsha, South Africa. AIDS 2004 4 9; 18(6). 10.1097/00002030-200404090-00006 15060436

[34] Ledergerber B, Lundgren JD, Walker AS, Sabin C, Justice A, Reiss P, et al Predictors of trend in CD4-positive T-cell count and mortality among HIV-1-infected individuals with virological failure to all three antiretroviral-drug classes. Lancet 2004 7 3–9; 364(9428). 10.1016/S0140-6736(04)16589-6 15234856

[35] Messele T, Abdulkadir M, Fontanet AL, Petros B, Hamann D, Koot M, et al Reduced naive and increased activated CD4 and CD8 cells in healthy adult Ethiopians compared with their Dutch counterparts. Clin Exp Immunol 1999 3; 115(3). 10.1046/j.1365-2249.1999.00815.x PMC1905237 10193416

[36] Kassu A, Tsegaye A, Wolday D, Wolday D, Hailu E, Sanders EJ, et al Role of incidental and/or cured intestinal parasitic infections on profile of CD4+ and CD8+ T cell subsets and activation status in HIV-1 infected and uninfected adult Ethiopians. Clin Exp Immunol 2003 4; 132(1). 10.1046/j.1365-2249.2003.02106.x PMC1808681 12653845

[37] Nakanjako D, Ssewanyana I, Mayanja-Kizza H, Kiragga A, Colebunders R, Manabe YC, et al High T-cell immune activation and immune exhaustion among individuals with suboptimal CD4 recovery after 4 years of antiretroviral therapy in an African cohort. BMC Infect Dis 2011 2 8; 11(43). 10.1186/1471-2334-11-43 PMC3065409 21299909

[38] Lederman MM, Kalish LA, Asmuth D, Fiebig E, Mileno M, Bush MP. ‘Modelling’ relationships among HIV-1 replication, immune activation and CD4^+^ T-cell losses using adjusted correlative analyses. AIDS 2000 5 26; 14(8). 10.1097/00002030-200005260-00006 10853976

[39] Hazenberg MD, Otto SA, van Benthem BH, Roos MT, Coutinho RA, Lange JM, et al Persistent immune activation in HIV-1 infection is associated with progression to AIDS. AIDS 2003 9 5; 17(13). 10.1097/00002030-200309050-00006 12960820

