# Supplementary material for: Low Incidence of HIV-1C Acquired Drug Resistance 10 Years after Roll-Out of Antiretroviral Therapy in Ethiopia: A Prospective Cohort Study
Source: PLoS One. 2015 Oct 29;10(10):e0141318. doi: 10.1371/journal.pone.0141318 (PMC4626118; doi:10.1371/journal.pone.0141318)
Supplement: S1 Table — Reprinted from BMC Infect Dis. 2014 Mar 22; 14:158. doi: 10.1186/1471-2334-14-158. (PDF) [file pone.0141318.s001.pdf]

Supporting Information

S1 Table: Baseline transmitted drug resistance in the RT gene among chronically infected patients from Northwest Ethiopia

Reprinted from BMC Infect Dis. 2014 Mar 22; 14:158. doi: 10.1186/1471-2334-14-158

Lab No.	Age/Sex	HIV load*	NRTI		NNRTI	
			Mutation	*Resistance to*	*Mutation*	*Resistance to*
5479	25 yrs/f	5.44	-	-	*E138G*	ETR
5480	35 yrs/f	4.20	-	-	**Y188H**	ETR, EFV, NVP
5905	35 yrs/f	4.58	**K219E**	AZT,D4T,ABC,DDI,TDF	-	-
5496	30 yrs/f	3.88	**L210W**	AZT,D4T	-	-
5489	28 yrs/f	5.76	-	-	*E138G*	ETR
5520	35 yrs/f	5.49	-	-	*E138A*	ETR
5616	30 yrs/f	4.31	**K65R**	ABC,ddI,FTC,3TC,d4T,TDF	-	-
5652	40 yrs/f	5.50	-	-	*E138A*	ETR
5763	45 yrs/f	4.91	-	-	*E138A*	ETR
5843	27 yrs/f	4.49	-	-	*E138A*	ETR
5501	27 yrs/f	4.38	-	-	*V90I*	ETR
5604	44 yrs/f	4.64	-	-	*V90I*	ETR
5491	25 yrs/m	5.94	-	-	**K101E**, *E138A*	EFV,ETR,NVP
5533	32 yrs/m	5.17	-	-	**G190A**	EFV,ETR,NVP,RPV
5566	30 yrs/m	5.49	-	-	*E138A*	ETR
5711	22 yrs/m	5.13	-	-	*E138A*	ETR
5727	35 yrs/m	4.80	-	-	*E138A*	ETR
5712	28 yrs/m	4.82	-	-	**G190A**	EFV,ETR,NVP,RPV
5710	38 yrs/m	5.17	**L210W**	AZT,D4T	-	-
5603	22 yrs/m	5.12	-	-	V90I	ETR
5991	24 yrs/m	4.36	-	-	**M230I**	RPV

*HIV load in $\log_{10}$ copies/ml; Mutations in bold are only considered by both IAS and Stanford University HIV drug resistance algorithm; Mutation in *Italics* are reported by IAS only.
Abreviations: *3TC* lamiduvine, *ddI* didanosine, *d4T* stavudine, *FTC* emtricitabine, *TDF* tenofovir, *ZDV* zidovudine, *NNRTI* non-nucleoside RT inhibitors, *EFV* efavirenz, *ETR* etravirine, *NVP* nevirapine, *RPV* rilpivirine.

Reprinted from [18] Mulu *et al. BMC Infectious Diseases* 2014 **14**:158 doi: 10.1186/1471-2334-14-158
